# Development and Evaluation of Rifampicin Loaded Alginate–Gelatin Biocomposite Microfibers

**DOI:** 10.3390/polym13091514

**Published:** 2021-05-08

**Authors:** Ameya Sharma, Vivek Puri, Pradeep Kumar, Inderbir Singh, Kampanart Huanbutta

**Affiliations:** 1 Chitkara College of Pharmacy, Chitkara University, Chandigarh 140401, India; ameya.sharma@chitkara.edu.in (A.S.); vivek.puri@chitkara.edu.in (V.P.); 2 Chitkara University School of Pharmacy, Chitkara University, Solan 174103, India; 3 Wits Advanced Drug Delivery Platform Research Unit, Department of Pharmacy and Pharmacology, School of Therapeutic Sciences, Faculty of Health Sciences, University of the Witwatersrand, Johannesburg 2193, South Africa; pradeep.kumar@wits.ac.za; 4 Faculty of Pharmaceutical Sciences, Burapha University, 169, Saensook, Muang, Chonburi 20131, Thailand

**Keywords:** biopolymers, microfibers, rifampicin, alginate, gelatin, nancoclay, xanthan

## Abstract

Various systematic phases such as inflammation, tissue proliferation, and phases of remodeling characterize the process of wound healing. The natural matrix system is suggested to maintain and escalate these phases, and for that, microfibers were fabricated employing naturally occurring polymers (biopolymers) such as sodium alginate, gelatin and xanthan gum, and reinforcing material such as nanoclay was selected. The fabrication of fibers was executed with the aid of extrusion-gelation method. Rifampicin, an antibiotic, has been incorporated into a biopolymeric solution. RF1, RF2, RF3, RF4 and RF5 were coded as various formulation batches of microfibers. The microfibers were further characterized by different techniques such as SEM, DSC, XRD, and FTIR. Mechanical properties and physical evaluations such as entrapment efficiency, water uptake and in vitro release were also carried out to explain the comparative understanding of the formulation developed. The antimicrobial activity and whole blood clotting of fabricated fibers were additionally executed, hence they showed significant results, having excellent antimicrobial properties; they could be prominent carriers for wound healing applications.

## 1. Introduction

The pervaded pathogens are abolished from an impaired or disrupted tissue site for efficacious repairing [1]. To evade infection and escalate the wound healing cycle, the wound site or damaged tissue site is protected, aided by sterile dressing material [2]. The three cascades such as inflammation, proliferation and tissue remodeling complete the wound healing process [3]. The topical application of an anti-microbial drug seems to be efficacious for healing or tissue repairing [4]. Rifampicin is an antibiotic drug particularly used against Gram-positive and Gram-negative bacteria and has exemplary antibacterial properties [5]. It is a liposoluble antibiotic, so penetration into most tissues is adequate. Rifampin is an antibiotic that inhibits DNA-dependent RNA polymerase activity in susceptible cells [6]. It is a suitable antibiotic to be encapsulated in microfibers for antimicrobial activity and it could be utilized for wound healing applications as well [7]. For drug delivery applications, fibrous structures, including microfibers and nanofibers, are deemed acceptable carriers [8,9]. The microfibers are reported as an attractive option because of their flexible and soft nature. The versatile composition of fibers comprising biopolymers and reinforcing material may serve as a wound dressing material. Wound exudates are also absorbed easily by the microfibers [10]. In the present research, biocomposite biopolymeric microfibers were fabricated using alginate, gelatin, xanthan gum, and nanoclay. The microfibers were prepared employing the ionotropic gelation method, and xanthan gum [11,12] and nanoclay [13,14] were additionally used to enhance the mechanical strength of the fibers. Furthermore, physicochemical, physico-mechanical, and morphological analyses were also executed. Within the polymeric matrix, microfibers displayed an intricate molecular interaction profile, providing an irregular drug release mechanism.

## 2. Materials and Methods

### 2.1. Materials

Sodium alginate, gelatin, calcium chloride and rifampicin were the reagents used. Rifampicin was obtained from Banson Pharmaceuticals as a gift sample. All the ingredients such as sodium alginate (CAS number—9005-38-3); xanthan gum (CAS number—11138-66-2); gelatin (CAS number—9000-70-8); nanoclay (CAS number—1332–58-7) and calcium chloride (CAS number—10035-04-8) were acquired from Sigma Aldrich, St. Louis, MO, USA. In the present research analysis, all analytical grade solvents, reagents and chemicals were employed.

### 2.2. Preparation of Rifampicin Loaded Biocomposite Microfibers

**Figure 1:**
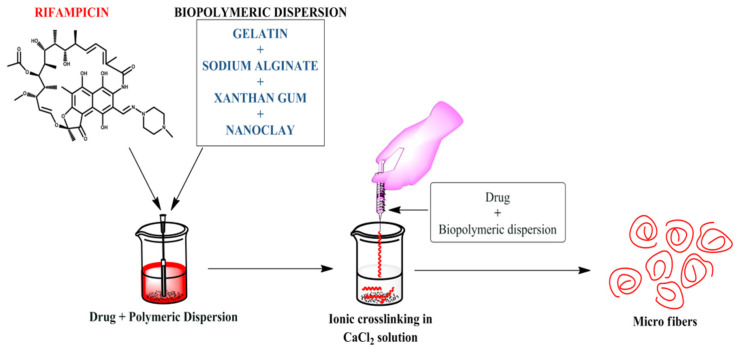
Fabrication method of rifampicin loaded biocomposite microfibers.

For the development of rifampicin biocomposite microfibers, the ionotropic gelation method was employed, as shown in Figure 1. In brief, as shown in Table 1, initially, the polymeric solution containing sodium alginate:gelatin solutions was prepared in weight ratios (5:5). To enhance the mechanical strength, xanthan gum and nanoclay were also added. The biopolymers (alginate and gelatin) were solubilized together by stirring at 500 rpm for 30 min, and an aqueous biopolymeric solution was prepared in which xanthan gum and nano clay were incorporated. For complete solubilization of gelatin, the biopolymeric dispersions were heated to 50 °C. First, rifampicin (50 mg) was dissolved in 5 mL of acetone and gradually incorporated into the aforementioned polymeric solution. This polymeric mixture (rifampicin + alginate + gelatin + xanthan gum + nanoclay) was then extruded into a beaker containing 1% calcium chloride (using a 22 gauge needle). The microfibers led to ionic cross-linking and were further washed with the help of water and dried (air or oven), as illustrated in Figure 1 [15,16,17].

### 2.3. Characterization of Rifampicin Loaded Biocomposite Microfibers

The morphological characteristics of rifampicin loaded biocomposite microfibers were assessed using entrapment efficacy, SEM analysis, and physicochemical analysis through FTIR and XRD. The physical properties were also executed as epitomized water uptake properties, mechanical characteristics, in vitro release and antimicrobial properties.

#### 2.3.1. Entrapment Efficiency

The cumulative amount of drug (rifampicin) which was entrapped in microfibers was calculated and weighed in ternion, employing the sample (length 10 cm). To extract the maximum drug from formulated microfibers, the sample was submerged in a solution (mixture of phosphate buffer pH 7.4 and ethanolic solvent in the ratio of 50:50 *v*/*v*) for over 24 h. In the presence of the hydro alcoholic solvent, the microfibers were crushed in the mortar and pestle; this solution was further strained and analyzed, aiding UV-Vis analysis (Perkin Elmer Lambda35) at 337 nm. Afterwards, a standard curve was plotted in ethanol using standard rifampicin solutions.

#### 2.3.2. Morphological Analysis

In order to study morphological characteristics, the biocomposite microfibers were adhered, aided by double sided adhesive tape, and placed carefully on a metallic stub. The developed fibers were sequentially plated with gold, and under various magnifications (250× and 500×) and at accelerating voltages (15 Kv), the surface of the fibers was visualized. The aforementioned scanning microscopy was performed on FEI Nova Nanolab 600 SEM, and to modify the samples acquired for clarity, brightness and contrast, Paint.net (graphic editor) was used.

#### 2.3.3. XRD

The X-ray diffractometry was carried out to investigate the crystallinity of rifampicin, rifampicin loaded fibers. Study was carried out using an X-ray diffractometer (Miniflex 2, Rigaku, Japan), which is a high potential and developed technique fundamentally having scattered intensity of an X-ray beam, which is hitting the sample as a function of incident and scattered angle, polarization and wavelength or energy. The flexibility and non-destructive operational protocols of the technique exhibit chemical composition, but this technique also shows the crystallographic structure of the raw samples. The microfibers were crushed in powder form and scanned from 0° to 80° diffraction angle (2Ɵ) range under the following measurement conditions: source, nickel filtered Cu-K radiation; voltage 35 kV; current 25 mA; scan speed 0.05 min^−1^.

#### 2.3.4. DSC

Thermal analysis was carried out employing differential scanning calorimetry with liquid nitrogen. The analysis was performed under purge of dry nitrogen gas. High purity indium was used to calibrate the heat flow and heat capacity of the instrument. Sample films of weight (2.5–5 mg) were placed in an aluminum cell and were firmly crimped with the lid to provide an adequate seal. The sample was heated at ambient temperature from 50 to 300 °C at a preprogrammed heating rate of 10 °C min^−1^.

#### 2.3.5. FTIR Studies

The spectroscopic analysis of biocomposite microfibers was performed to determine inter molecular interactions in between the drug, constituent biopolymers and reinforcing material employing (IFS66/S, Alpha Bruker, Leipzig, Germany). This study was carried out in the region of 4000–650 cm^−1^ wave number in the reflectance mode.

#### 2.3.6. Water Uptake

The water uptake property of the biocomposite microfiber polymeric matrix was studied and calculated at pH 7.4 phosphate buffer saline. The formulated microfibers were weighed (the initial weight) and then submerged for 24 h in the aforementioned PBS (10 mL) prior to being deliberately removed, aided by a tweezer or plucker. With the help of filter paper, water present on the surface was gradually soaked up and the wet weight referred to as final weight was acquired.

% Water uptake was determined using the following equation:

$$\mathrm{Water}\;\mathrm{uptake}=\left[\frac{\mathrm{Initial}\;\mathrm{weight}-\mathrm{Final}\;\mathrm{weight}}{\mathrm{Initial}\;\mathrm{weight}}\right]\times 100$$

#### 2.3.7. Mechanical Properties

The mechanical characteristics of biocomposite microfibers were evaluated, aided by a uniaxial tensile machine. The tensile machine (Instron5943, Canton, MA, USA) was held and loaded with a cell size of 10N with specified cross head speed (5 mm/min). The biocomposite microfiber samples were sliced into a specified length of 5 cm and, further, this extension test was conducted at room temperature. The following equations were then used to calculate tensile strength and elongation at break.

$$\mathrm{Tensile}\;\mathrm{strength}=\frac{\mathrm{Breaking}\;\mathrm{force}\;\left(N\right)}{\mathrm{cross}-\sec\mathrm{tional}\;\mathrm{area}\;\mathrm{of}\;\mathrm{sample}\;\left({\mathrm{mm}}^{2}\right)}\;\mathrm{Elongation}\;\mathrm{to}\;\mathrm{break}=\frac{\mathrm{Increase}\;\mathrm{in}\;\mathrm{length}\;\mathrm{at}\;\mathrm{breaking}\;\mathrm{point}\;\left(\mathrm{mm}\right)}{\mathrm{original}\;\mathrm{length}\;\left(\mathrm{mm}\right)}\times 100$$

#### 2.3.8. In Vitro Drug Release Studies

The drug release pattern of biocomposite microfibers was executed at 37 °C for 72 h in phosphate-buffered saline (PBS; pH 7.4). The microfibers (5 mg rifampicin) were poured into PBS (50 mL) in a sealed container and mounted on an orbital shaker machine. Thereafter, aliquots of 3 mLwere extracted at fixed time intervals such as 0.5, 1, 2, 4, 8, 12, 24, 48 and 72 h. All the samples were subsequently replaced with fresh PBS to perpetuate volume. The sample microfibers were removed and then analyzed at 337 nm. To analyze the drug release mechanism, the data profile was countered into varied models such as zero order, first order, Higuchi matrix, Hixson–Crowell, and Korsmeyer–Peppas release data models—kinetic experiments were then carried out.

Zero order—$Q=Q_{0}+k_{0}\;t$
First order—$\ln Q=\ln Q_{0}+k_{1\;}t$
Higuchi model—Q=kHt12
Hixson–Crowell model—Q013−QR13=ks t
Korsmeyer–Peppas model—$Q/Q_{T}=k_{kp\;}t^{n}$

Depict:

*Q* = total amount of drug release at time *t*;
*Q*_0_ = initial amount of drug;
*Q_R_* = amount of remaining drug at time *t*;
*Q_T_* = cumulative amount of drug release;
*k*_0_, *k*_1_, *k_H_*, *k_s_* and *k_kp_* = kinetic constants for all respective models such as zero order, first order, Higuchi, Hixson–Crowell and Korsmeyer–Peppas models;
n = release exponent.

#### 2.3.9. Whole Blood Clotting

The fibers were placed into polypropylene tubes and the aforementioned tubes were pre-warmed to 37 °C. Citrated whole blood (0.2 mL) was then slowly dispersed onto the fibers and then 0.2 M of CaCl_2_ solution (20 µL) added to start coagulation. The tubes containing blood samples were incubated at 37 °C + 30 rpm for 10 min. After 10 min, red blood cells that are free and are not trapped in the clot were hemolyzed with 25 mL of water and the absorbance of the resulting hemoglobin solution was measured at 540 nm.

#### 2.3.10. Antimicrobial Studies

The agar disc method was employed in order to assess the antimicrobial activity of the formulated biocomposite fibers. The two bacterial strains such as *Staphylococcus aureus* (Gram-positive bacterium) and *Escherichia coli* (Gram-negative bacterium), primarily present in the wound bed, were used in this process. The solution for agar was developed using the technique using Hi-Media. Briefly, 28 g of agar powder was dispersed in 1000 mL of filtered water. The solution was heated to boil so that the medium was fully dissolved and further sterilized by autoclaving at 121 °C for 15 min. Cooling of up to 40–50 °C was allowed, and by pouring 20 mL of liquid agar media, the agar plates were prepared. The bacterial culture suspension of *S. aureus* and *E. coli* was inoculated in large Petri dishes to initiate rapid growth of microorganisms. To become solidified, Petri plates were retained and a 6 mm pit was created, plying a sterilized borer. The drug-containing control group (rifampicin) and rifampicin fiber (RF5) were mounted in an agar plate pit and incubated for 24 h at 37 °C, with a deliberate inhibition zone.

## 3. Results and Discussion

**Figure 2:**
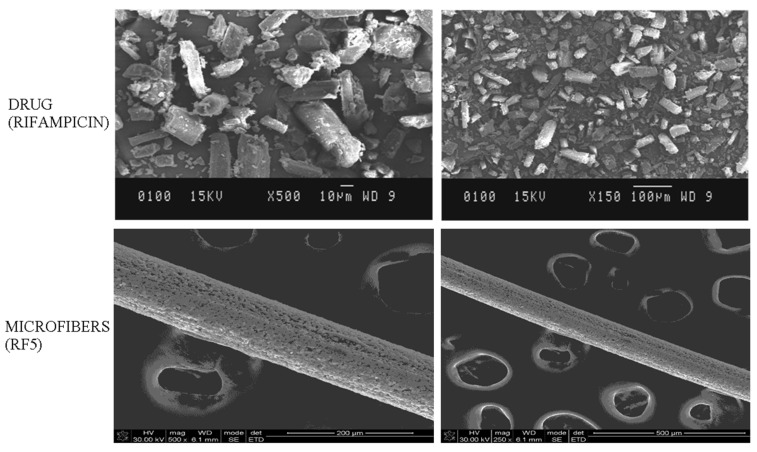
Morphological analysis of drug (rifampicin) and microfibers (RF5) at magnification value at 500× and 150×.

The entrapment efficiency of rifampicin loaded microfibers was found to be 91.14 ± 3.23% for RF1 and 96.34 ± 1.76% for RF5 microfibers, as seen in Figure 5 and Table 2. Contrastingly, the entrapment efficiency was improved with an inclusion of gelatin in microfibers, which could be attributed to partial inter molecular interactions between the drug (rifampicin) and gelatin. RF3′s entrapping efficiency was 95 ± 2.31%, which may be attributed to xanthan gum incorporation. The microfibers’ (RF5) entrapment efficiency was found to be the highest because of the addition of xanthan gum and nanoclay, which may be due to enriched interaction between the polymeric molecules and reinforcing material. SEM was used to study the surface morphology of alginate/gelatin biocomposite microfibers. Between 200 and 400 μm, the diameter of the microfibers was determined. On the surface of the microfibers, crystal deposition was visualized clearly, which could be attributable due to the precipitation of calcium chloride crystals after deposition to the surface during the process of drying. A smooth homogeneous morphology was revealed on the surface that clearly demonstrated augmented miscibility and consistent homogeneity between the polymers, as shown in Figure 2.

**Figure 3:**
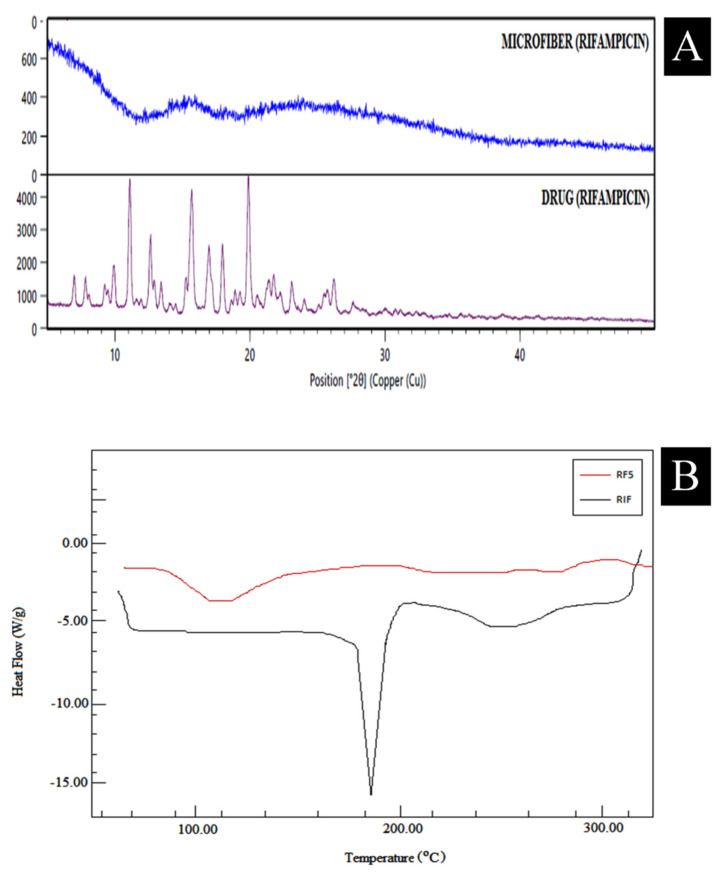
(**A**) XRD spectra of drug (rifampicin) and microfiber (RF5), (**B**) DSC thermogram of drug (rifampicin) and microfiber (RF5).

Rifampicin has a crystalline nature and exhibits major peaks at 2θ = 11.1, 12.7, 15.8, 16.3, 17.2, 18.1, and 20, respectively, according to the effects of the XRD pattern. In fabricated microfiber, the loss of crystalline peaks was observed (RF5). The results showed intercalation and potential bonding between the polymers, thus indicating that the drug was effectively trapped between the polymeric matrix in the microfibers, as depicted in Figure 3a. To scrutinize the interaction of polymers with the crystalline nature of entrapped rifampicin in the polymeric matrix of microfibers, and further embedded into the reservoir system of transdermal films, DSC analysis was performed and DSC curves are depicted in Figure 3b. The thermogram suggested that rifampicin showed an endothermic sharp peak at 183.7 °C whereas this prominent peak was found to be absent, which confirmed that the drug was molecularly dispersed into the matrix of rifampicin microfibers. It also confirmed its existence in an amorphous form.

**Figure 4:**
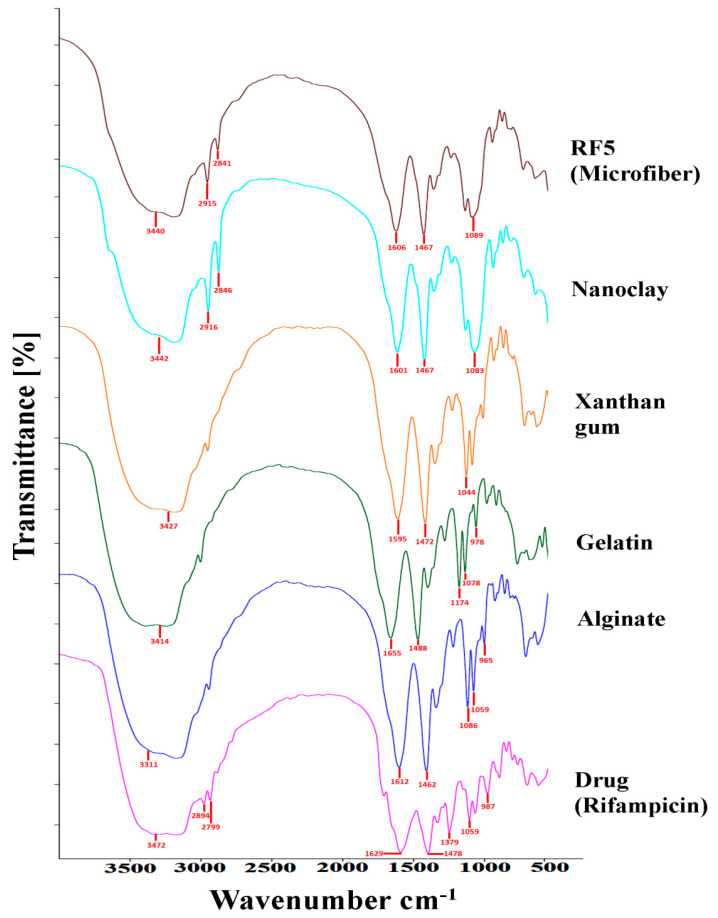
FTIR spectra of drug (rifampicin), alginate, gelatin, nanoclay and microfiber (RF5).

In order to provide accurate information on the interaction seen between formulations, Fourier transform infrared (FTIR) attenuated total reflectance was used. The FTIR spectra of the drug (rifampicin), formulated rifampicin microfibers and polymers used are shown in Figure 4. The FTIR drug rifampicin spectrum showed a peak of 3472 cm^−1^ (NH stretching), 2894 cm^−1^ (C-H bonding), 1629 cm^−1^ (C=O), 1478 cm^−1^ (C=C), 1379 cm^−1^ (CH2, C=C), 1059 cm^−1^ (-CH, CO, C-H), and 987 cm^−1^ (≡C-H, C-H) [18]. The IR range of alginate had characteristic absorption peaks at 3311 cm^−1^, which showed OH stretching, 1612 cm^−1^, which showed carboxylic C=O, 1298 cm^−1^, which possessed C-CH, 1086 and 1059 cm^−1^, ascribed to C-O stretching, 1032 cm^−1^, which represented C-C, 942 cm^−1^, which showed C-O, 888 cm^−1^, which manifested CH, and 822 cm^−1^, which represented Na-O [19]. The substantial gelatin peaks showed absorption bands at 3414 cm^−1^, confirmed NH stretching, 1655 cm^−1^, confirmed amide C=O, 1556 cm^−1^, proclaimed amide NH bending, and 1343 cm^−1^, ascribed to C-N stretching [20]. The prominent peaks of xanthan gum showed peaks at 3427cm^−1^ (O-H stretching), 2995 cm^−1^ (aliphatic C-H stretching), 1635 cm^−1^ (COO symmetric stretching), 1434 cm^−1^ (asymmetric stretching) and 1245–1043 cm^−1^ (pyranoid C-O-C ring stretching) [21]. The FTIR spectra of nanoclay exhibited peaks at 3442 cm^−1^ (OH stretching), 2916–2846 cm^−1^ (methylene symmetric vibration), 1737–1467 cm^−1^ (C=O, C-O-H stretching and hydrogen bonds), and 1063 cm^−1^ (Si-O bending) [22]. The FTIR spectrum relating to microfibers was similar to the polymer spectrum. Due to the lack of optimum peak characteristics of rifampicin in the FTIR spectra of microfibers as they have been cloaked by bands formed by polymers, it could be inferred that rifampicin was well embedded in the microfibers [17].

**Figure 5:**
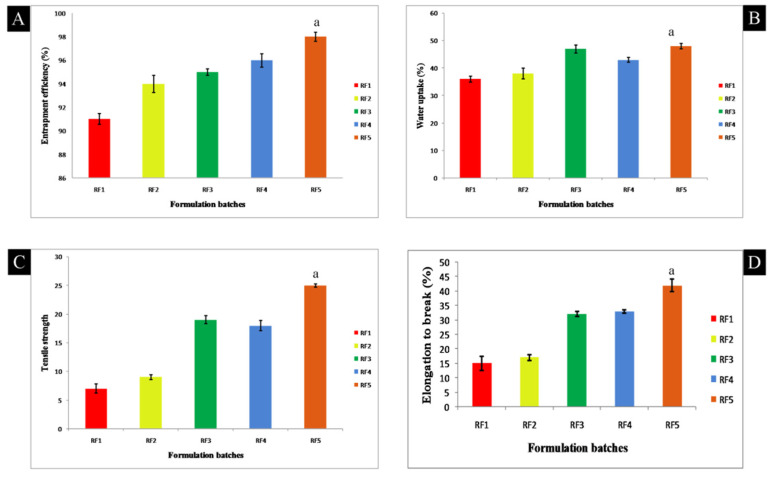
Bar graphs of different parameters (**A**) entrapment efficiency; (**B**) water uptake; (**C**) tensile strength (**D**) elongation to break (data were presented as mean ± SD and analyzed by one-way ANOVA followed by Tukey’s test as post hoc analysis. ‘a’ represents *p* < 0.05 vs. RF1, RF2, RF3 and RF4).

In developed microfibers, the water uptake property or capability of alginate–gelatin biocomposite microfibers was found to be enhanced; improved network formation was as imparted by gelatin concentration. In addition to xanthan gum and nanoclay, the percentage of water absorption of biocomposite microfibers was decreased, with a maximum value of 47.24 ± 3.28% in the case of RF5 (Figure 5 and Table 2). As rifampicin is a lipophilic drug, its prevalence in the biopolymer matrix may be due to this reduction in water affinity.

Pertaining to mechanical parameters, tensile strength and elongation to break (extensibility) were evaluated. Tensile strength and break elongation differed between 7.12 ± 0.25 to 25.28 ± 0.76 N/mm^2^ and 15.20 ± 0.98% to 42.09 ± 1.09%. With the addition of xanthan gum and nanoclay, the tensile strength of the microfibers improved as illustrated in Figure 5 and Table 2. An increase in the mechanical strength of biocomposite fibers may be due to the association and bond formation between the biopolymers used. The addition of nanoclay has significantly enhanced this mechanical property. This may be due to the reinforcing material’s capability to act as fillers (rigid) in the polymeric matrix, as well as intermolecular interactions between the polymeric matrix and layered silicates through H bonds. As explained in the FTIR discussion, the tensile strength in blended microfibers is effectively enhanced due to the partial contact between the biomacromolecules [20].

**Figure 6:**
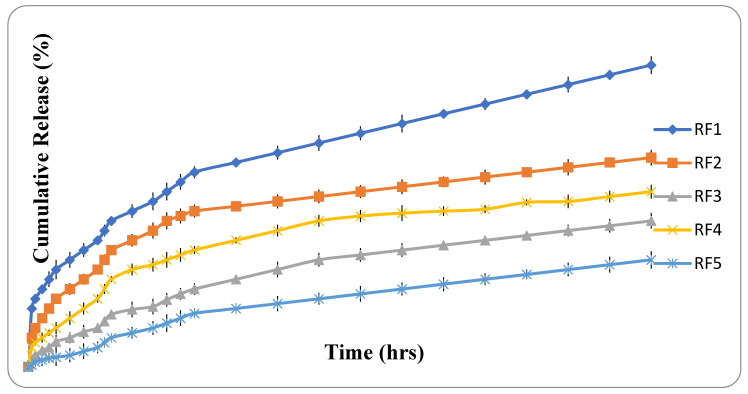
In vitro release of various formulation batches of microfibers.

The results of the in vitro drug release study revealed that microfibers containing a combination of polymeric content and reinforcing material resulted and thereby minimized the total percent release of the drug. The drug releases from RF1, RF2, RF3, RF4 and RF5 were observed to be 31.28 ± 4.36%, 20.23 ± 3.12%, 14.06 ± 2.2%, 17.22 ± 3.08% and 9.03 ± 2.04%, respectively, in 90 h, as depicted in Figure 6. Alginate polymer matrix formation may be a retardant factor in the release of the drug from prepared fibers. Furthermore, the incorporation of xanthan gum and nanoclay delayed drug release from prepared microfibers. This may be due to the formation of intermolecular hydrogen bonds between polymeric chains and reinforcing material in biocomposite fibers. Molecular interaction with polymeric chain relaxation (up to hydration) could also lead to a decrease in the release of the drug from RF5. The drug release data obtained after in vitro release study were further analyzed via fitting into various kinetic models, i.e., zero order, first order, Higuchi equation, Hixson–Crowell model, and the power law. The coefficient of determination (*r*^2^) was much closer to 1 for the first order equation. All the formulations followed non-Fickian diffusion with *n* values falling within the 0.5 < *n* < 1.0 range and depicted anomalous release behavior as shown in Table 3. This value indicates a combination of diffusion and erosion controlled mechanisms responsible for the release of the drug from the biocomposite fibers.

**Figure 7:**
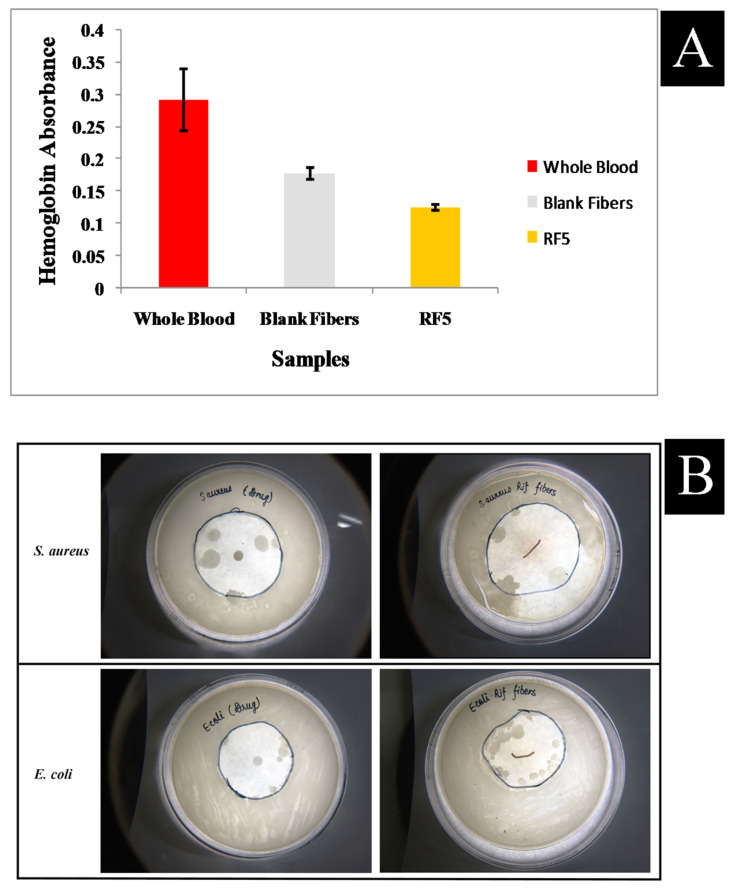
(**A**) Whole blood clotting of whole blood, blank microfibers and rifampicin loaded microfibers, (**B**) Antimicrobial activity of standard group (Rifampicin) and microfibers (RF5) against *Staphylococcus aureus* and *Escherichia coli*.

In order to evaluate whether blank fibers and rifampicin loaded fibers (RF5) can increase the rate of blood clotting, whole blood was contacted with fibers for 10 min before hemolyzing RBCs that were not trapped in the clot that formed on the fiber surface. A higher absorbance value of the hemoglobin solution thus indicates a slower clotting rate. Rifampicin loaded fibers led to significantly lower absorbance values than blank fibers, as shown in Figure 7a. The antimicrobial activity of rifampicin against bacteria (Gram-positive and Gram-negative bacteria) was assessed using the disc diffusion method. The zone of inhibition of microfiber (RF5) was found to be 23 mm against *S. aureus* and 22 mm against *E. coli*. In comparison to the normal or control group, rifampicin had an inhibition zone of 24 mm against *S. aureus* and 20 mm against *E. coli*. The antimicrobial study demonstrated that the drug was effectively released from the microfiber polymer matrix and exhibited antimicrobial activity, which can be seen in Figure 7b.

## 4. Conclusions

An appropriate analytical method for rifampicin was developed by the UV/Visible spectrophotometer, and rifampicin showed maximal absorption at a wavelength of 337 nm in a solution mixture comprised of phosphate buffer saline (pH 7.4) and ethanol in the ratio (50:50, *v*/*v*). The formulation produced was identified by different techniques such as SEM, XRD, DSC and FTIR. The addition of xanthan gum and nanoclay relatively enhanced the mechanical strength individually, and when used in the combination with alginate and gelatin, it significantly increased the mechanical properties. The kinetic analysis was conducted and fitted into various kinetic models in which the anomalous behavior of all formulations produced was represented by n value. As a controlled drug delivery system, the formulation batches have maximum release at 90 h, implying the prolonged retardation of drug release from the prepared microfibers. Since biocomposite microfibers have good mechanical properties, they may be an effective candidate for developing a controlled drug delivery system and prolonged drug delivery system. The microfibers could be potential candidates and effective biopolymeric wound dressing materials for wound healing applications, as they implied excellent anti-microbial properties.

## Figures and Tables

**Table 1:** Formulation composition of rifampicin biocomposite microfibers.

Formulation Batches	Sodium Alginate (%)	Gelatin (%)	Xanthan Gum (%)	Nanoclay (%)	Rifampicin (mg)
RF1	2	-	-	-	50
RF2	2	2	-	-	50
RF3	2	2	0.5	-	50
RF4	2	2	-	0.5	50
RF5	2	2	0.5	0.5	50

**Table 2:** Various parameters of formulation batches.

Formulation Code	Entrapment Efficiency (%)	Water Uptake (%)	Tensile Strength (N/mm^2^)	Elongation to Break (%)
RF1	91.14 ± 3.23	36.42 ± 1.22	7.12 ± 0.25	15.20 ± 0.98
RF2	94.34 ± 1.24	38.16 ± 2.18	9.44 ± 0.73	17.46 ± 0.42
RF3	95.21 ± 2.31	47.11 ± 2.14	19.23 ± 0.72	32.27 ± 1.06
RF4	94.97 ± 2.17	42.52 ± 2.64	18.21 ± 0.78	33.34 ± 1.07
RF5	96.34 ± 1.76	47.24 ± 3.28	25.28 ± 0.76	42.09 ± 1.09

**Table 3:** Drug release kinetic modeling data of various microfiber batches.

Batches	Zero Order		First Order		Higuchi Model		Hixson Crowell Model		KorsmeyerPeppas Model		
	*r* ^2^	*k*_o_	*r* ^2^	*k*_1_	*r* ^2^	*k*_H_	*r* ^2^	*k*_HC_	*r* ^2^	*K*_kp_	*N*
RF1	0.877	0.271	0.980	−0.012	0.984	2.922	0.972	−0.020	0.988	0.817	0.334
RF2	0.780	0.190	0.970	−0.014	0.942	2.131	0.939	−0.019	0.981	0.616	0.388
RF3	0.928	0.160	0.992	−0.013	0.944	1.683	0.993	−0.018	0.994	0.061	0.392
RF4	0.832	0.177	0.989	−0.015	0.965	1.947	0.965	−0.020	0.977	0.388	0.424
RF5	0.951	0.121	0.983	−0.011	0.986	1.254	0.988	−0.015	0.985	0.403	0.469

## Data Availability

Not applicable.
